# Experimental demonstration of a diamond quantum vector magnetometer for deep-sea applications

**DOI:** 10.1093/nsr/nwae478

**Published:** 2025-01-15

**Authors:** Ziyun Yu, Yunbin Zhu, Wenzhe Zhang, Ke Jing, Shuo Wang, Chuanxu Chen, Yijin Xie, Xing Rong, Jiangfeng Du

**Affiliations:** CAS Key Laboratory of Microscale Magnetic Resonance and School of Physical Sciences, University of Science and Technology of China, Hefei 230026, China; Anhui Province Key Laboratory of Scientific Instrument Development and Application, University of Science and Technology of China, Hefei 230026, China; CAS Key Laboratory of Microscale Magnetic Resonance and School of Physical Sciences, University of Science and Technology of China, Hefei 230026, China; Anhui Province Key Laboratory of Scientific Instrument Development and Application, University of Science and Technology of China, Hefei 230026, China; CAS Key Laboratory of Microscale Magnetic Resonance and School of Physical Sciences, University of Science and Technology of China, Hefei 230026, China; Anhui Province Key Laboratory of Scientific Instrument Development and Application, University of Science and Technology of China, Hefei 230026, China; CAS Key Laboratory of Microscale Magnetic Resonance and School of Physical Sciences, University of Science and Technology of China, Hefei 230026, China; Anhui Province Key Laboratory of Scientific Instrument Development and Application, University of Science and Technology of China, Hefei 230026, China; Institute of Deep-sea Science and Engineering, Chinese Academy of Sciences, Sanya 572000, China; Institute of Deep-sea Science and Engineering, Chinese Academy of Sciences, Sanya 572000, China; Institute of Quantum Sensing and School of Physics, Zhejiang University, Hangzhou 310027, China; CAS Key Laboratory of Microscale Magnetic Resonance and School of Physical Sciences, University of Science and Technology of China, Hefei 230026, China; Anhui Province Key Laboratory of Scientific Instrument Development and Application, University of Science and Technology of China, Hefei 230026, China; CAS Key Laboratory of Microscale Magnetic Resonance and School of Physical Sciences, University of Science and Technology of China, Hefei 230026, China; Anhui Province Key Laboratory of Scientific Instrument Development and Application, University of Science and Technology of China, Hefei 230026, China; Institute of Quantum Sensing and School of Physics, Zhejiang University, Hangzhou 310027, China

**Keywords:** quantum sensing, deep-sea sensor, nitrogen-vacancy center, vector magnetometry

## Abstract

Magnetometry plays an important role in exploring the deep sea, which is one of the Earth’s final unknown frontiers. However, the complexity of the marine environment and the limitations of conventional magnetometers restrict its in-depth application. The nitrogen-vacancy (NV) center in diamond offers a potential solution to encompass and transcend conventional ocean magnetometers. Its unique advantages, such as precise vector measurement and tolerance to extreme environments, make it well suited for deep-sea applications like navigation. This work introduces the first deep-sea quantum vector magnetometer based on NV centers. The performance of this magnetometer is effectively validated by a series of field tests on the manned submersible Shenhai Yongshi during a cruise in the South China Sea, including an experimental underwater navigation using the diamond quantum sensor as a magnetic compass. This successful deep-sea application marks a milestone for transforming this promising solid-state spin quantum system into a practical sensor for real-world marine applications.

## Teaser

The first deep-sea experimental application of diamond quantum vector magnetometer.

## INTRODUCTION

The ability of magnetometry to utilize Earth’s magnetic field and identify weak magnetic signals is essential in marine exploration. This technology has been widely applied across many scientific and engineering domains, including mineral exploration [1], magnetic mapping [2], underwater navigation [3], geophysical research [4] and volcano observation [5]. The history of ocean magnetometry dates back to the 1950s. Early ocean magnetometers were primarily based on a fluxgate sensor [6], and modern magnetometers tend to employ various sensing technologies for more specific applications, such as the proton precession magnetometer [6], the atomic vapor cell magnetometer [7], the superconducting quantum interference device (SQUID) [8] and the microelectromechanical systems (MEMS) magnetometer [9]. For a typical underwater magnetic survey, the magnetometers need to perform measurement at their own spatial positions, so various types of underwater vehicles are employed as movable ocean sensor platforms, including ship-towed vehicles [10,11], underwater gliding vehicles [7], remotely operated vehicles [12], autonomous underwater vehicles [13] and manned submersibles. The wide-range applications and sensor platforms in marine environments necessitate the selection of different marine magnetometers based on specific requirements.

Marine scalar magnetometers have been well developed and widely used in ocean magnetometry. These magnetometers measure the total magnitude of the magnetic field without directional information. Atomic vapor cell magnetometers are distinguished among these sensors for their high sensitivity, which have been increasingly used in oceanographic and geophysical applications in recent years [15]. Meanwhile, performing full-vector magnetometry on moving platforms still appears to be challenging [16,17]. The resource consumption and system complexity strictly limit the high-sensitivity magnetometers like SQUID [8,18]. The MEMS magnetometers and fluxgate magnetometers have the advantage of low power consumption and miniaturized size, but the relatively low sensitivity constrains their broader adoption [9,19]. Moreover, full-vector measurement based on these magnetometers cannot normally be achieved using a single sensor. Instead, it requires the integration of multiple sensors, each positioned at different locations and orientations, which is sensitive to slight attitude changes and sensor vibrations. This setup introduces systematic errors such as misalignment and inconsistency, which limits the full-vector magnetometry in practical applications [19,20]. Besides, ocean magnetometers also face general challenges such as high hydrostatic pressure, varying attitude in the Earth’s magnetic field and electromagnetic noise from the vehicle itself [10].

Facing these challenges, the nitrogen-vacancy (NV) center in diamond emerges as a competitive candidate of the next-generation general ocean magnetometer. The diamond lattice provides intrinsic directional reference that allows for full-vector measurements with a single sensor, thereby overcoming the common challenge faced by vector magnetometers [21,22]. The diamond NV center also possesses high dynamic range [23], high sensitivity [24,25] and compatibility in extreme environments [26]. These features position it as a well-suited sensor for full-vector marine magnetometry. Compared to atomic vapor cell magnetometers, diamond magnetometers could directly provide directional information of the magnetic field with a single sensor. Besides, the diamond magnetometers do not have the dead zone that disables the output in certain orientations [15], which is a common issue for vapor cell magnetometer devices in marine sensing. Research on atomic vapor cell magnetometers has led to some laboratory setups that achieve similar capabilities with increased system complexity [27,28], but they have not been used in applications like marine sensing. Furthermore, the solid-state feature of NV center sensors allows for a potentially miniaturized design on chip [29], enabling their compact integration into various underwater vehicles without the severe trade-off between performance, power consumption and size in conventional magnetometers.

**Figure 1.**
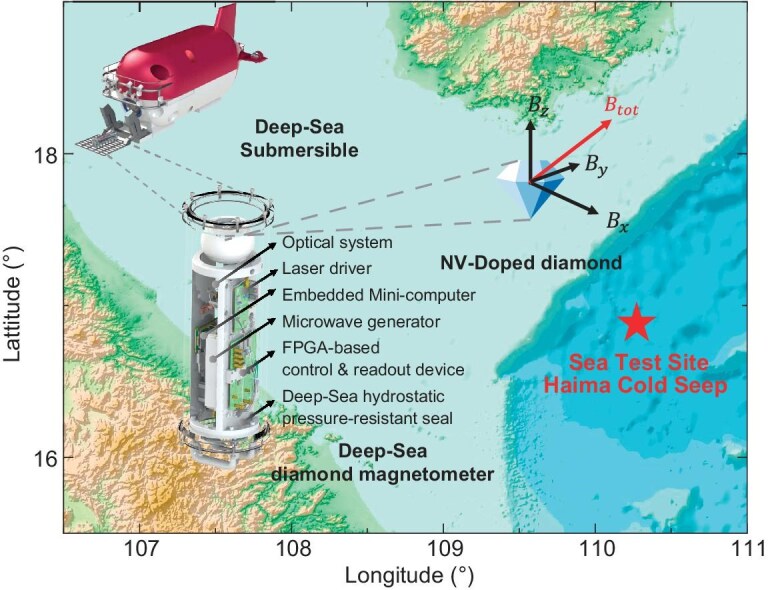
Overview of the diamond ocean magnetometer and deep-sea experiments. The figure showcases a deep-sea exploration in the South China Sea using the full-vector magnetometer based on a diamond NV center. The red icon in the figure denotes the experiment’s diving site, known as the Haima Cold Seep. In this setup, the diamond magnetometer is mounted on a deep-sea submersible and works as a realtime sensor. The device incorporates a suite of integrated subsystems designed for the control and readout of the quantum states of diamond NV centers, all housed within a robust, cylindrical, watertight compartment crafted from titanium alloy. The bathymetric data are obtained from the GEBCO grid [14].

In this work, we report a quantum sensor using NV centers in diamond for deep-sea applications, as shown in Fig. 1. The diamond quantum magnetometer is installed on a manned deep submersible vehicle named Shenhai Yongshi in the strapdown configuration [30]. During a cruise in the South China Sea, the device’s capability of dynamic vector magnetometry was validated through a series of field tests. In the development of the device, we craft a compact quantum control and readout system for the NV center, and integrate the system into independent functional modules. These components, along with the diamond probe and electronic devices, are encapsulated within a watertight cylinder. This sensor integration is specifically designed for deep-sea full-vector magnetometry applications. An integrated optical system with light-trapping diamond waveguide is designed to excite the NV centers and effectively collect the fluorescence carrying physical quantity information [31]. A multi-channel microwave system is developed to manipulate the NV centers aligning along all four crystallographic axes in the diamond lattice. Quantum states of the NV centers along these directions are readout simultaneously using a frequency-division multiplexing scheme and a proportional–integral–derivative (PID) frequency-locking algorithm. A miniaturized lock-in amplifier device developed in previous work is employed in the system [32]. By further exploiting the potential of this innovative quantum sensor, we could extend our capabilities in underwater exploration and elevate our understanding of the ocean’s depths to a new level in the future.

## DIAMOND MAGNETOMETRY

The NV center is a photoluminescent defect in the diamond lattice, composed of a nitrogen atom substituting a carbon site and an adjacent vacancy in the lattice structure [33–35]. The negatively charged NV center is sensitive to multiple physical quantities and can be readout by an optically detected magnetic resonance (ODMR) method. The extraordinarily long spin lifetime of the NV center at room temperature makes it a unique choice for quantum information and quantum sensing [24]. These features of the NV center position it as a promising magnetometer, offering high sensitivity and intrinsic crystallographic axes for full-vector measurement capability [36–38]. The stable property of diamond also allows it to work in extreme environments [39].

**Figure 2.**
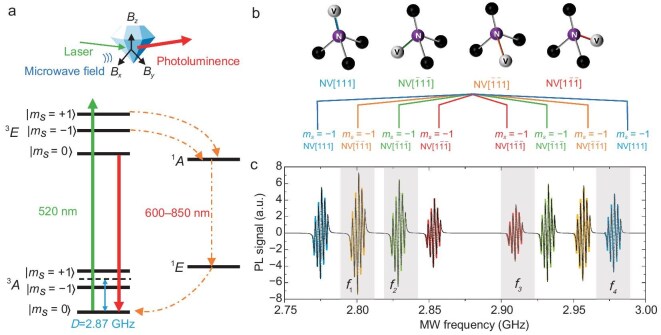
Schematic of vector magnetometry performed by the NV centers in diamond. (a) Energy level diagram of the NV center in diamond. The zero-field splitting $D_{\it gs}$ denotes the energy gap between the ground electronic spin levels $\mathinner {|{m_s=0}\rangle }$ and $\mathinner {|{m_s=\pm 1}\rangle }$. When a magnetic field is applied, the degeneracy of the $\mathinner {|{m_s=\pm 1}\rangle }$ states is lifted, causing Zeeman splitting, which enables vector magnetic field sensing. A 520-nm green laser can be used to excite the NV center from the ground state to the excited state, while the resultant red photoluminescence (PL) from spontaneous radiation serves as an optical readout for magnetic resonance. By applying a microwave field that matches the NV center’s transition frequency, its state can be manipulated. (b) The four crystalline structure figures demonstrate the different NV centers along all four crystallographic orientations. (c) The first-order derivative ODMR spectrum of the NV centers obtained by simultaneous microwave (MW) frequency sweeping with different orientations, each denoted by corresponding colors as in (b). The black line represents the fitted spectrum using Lorentzian profile. The hyperfine energy levels are simultaneously manipulated to enhance the signal contrast. The four resonant frequencies $f_{1\!-\!4}$ utilized in full-vector magnetometry are highlighted in the figure with a gray background.

As demonstrated in Fig. 2a, the NV center features a ground-state electronic spin in the form of triplet $^3A$. The energy levels $\mathinner {|{m_s=0}\rangle }$ and $\mathinner {|{m_s=\pm 1}\rangle }$ are split by zero-field splitting, $D_{\it gs}\approx 2.87\ \text{GHz}$. This splitting results from spin-spin interactions within the color center and is dependent on temperature. The effective Hamiltonian describing the NV center’s spin system is given by


\begin{eqnarray*}
H_{\it eff}=D_{\it gs}hS_z^2+g\mu _B\vec{B}_0\cdot \vec{S},
\end{eqnarray*}

where $\vec{S}=(S_x,S_y,S_z)$ is the dimensionless electronic spin-1 operator with $\hat{z}$ parallel to the NV axis, $g$ is the electronic $g$ factor of the NV center and $\mu _B$ is the Bohr magneton.

In the presence of an external magnetic field $\vec{B}_0$ aligned with the direction of the NV center, the transition between $\mathinner {|{m_s=\pm 1}\rangle }$ and $\mathinner {|{m_s=0}\rangle }$ experiences a Zeeman splitting and the degeneracy is lifted. By measuring both the transition frequencies of $\mathinner {|{m_s=0}\rangle }\leftrightarrow \mathinner {|{m_s=+1}\rangle }$ and $\mathinner {|{m_s=0}\rangle }\leftrightarrow \mathinner {|{m_s=-1}\rangle }$, the magnetic field and zero-field splitting can be simultaneously extracted. The temperature dependence of zero-field splitting $dD_{\it gs}/dT\approx -74\;\ \text{kHz/K}$ can also be utilized to detect the temperature change from the transition frequencies [40,41]. After the NV center is excited to state $^3 E$ by a 520-nm green laser as in Fig. 2a, its spin state can be optically readout by the red fluorescence emitted during the NV center’s spontaneous emission to the ground state. The NV center emits red fluorescence at a higher rate in the $\mathinner {|{m_s=0}\rangle }$ state compared to the $\mathinner {|{m_s=\pm 1}\rangle }$ states. This is due to the increased probability of non-radiative transitions through the intermediate singlet states $^1A$ and $^1E$ at the $\mathinner {|{m_s=\pm 1}\rangle }$ states. Therefore, the quantum states of this spin system can be identified from the intensity of the red fluorescence.

To implement vector magnetometry with a diamond sensor, it is necessary to separately extract the photoluminescence (PL) signal from the NV centers oriented along different crystallographic axes in the diamond lattice. Here we adopt a frequency-division multiplexing scheme based on microwave modulation technology. [21,42,43] The applied microwave field resonant with different NV centers is frequency modulated by corresponding preset square waves, which presents as a periodical change of the fluorescence signal in measurement. A photodiode is used to collect the fluorescence, and the resulting photocurrent is subsequently demodulated to extract the spin-state information of the four differently oriented NV centers from the overall photocurrent. The crystal structure of the NV centers is depicted in Fig. 2b, showcasing the diamond’s face-centered-cubic crystal symmetry. By applying an external bias magnetic field with appropriate projections along all diamond crystallographic axes, it is possible to separate the resonant frequencies, thereby enabling simultaneous NV center manipulation. As demonstrated in Fig. 2b, the energy diagram represents the NV centers aligning along four orientations in the presence of an external magnetic field of about $5\ \text{mT}$. As an example, a continuous-wave optically detected magnetic resonance (CW-ODMR) spectrum of the NV color centers in the diamond ensemble is presented in Fig. 2(c), which includes eight sets of resonance frequencies from NV centers in four orientations. Among these resonant frequencies, four frequencies $f_1, f_2, f_3, f_4$ along different crystallographic axes are selected to perform vector measurement and suppress common-mode temperature drift.

**Figure 3.**
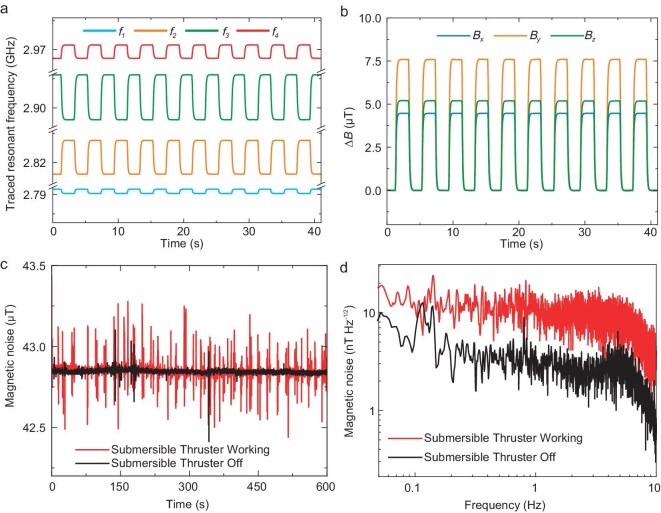
The performance test of the diamond magnetometer. (a) The time-domain waveform of the resonant frequency response in the presence of a square-wave magnetic field. Each line represents a different orientation of the NV centers in diamond, as indicated in Fig. 2(c). (b) The time-domain waveform of the external magnetic field in (a) calculated from the four NV resonant frequencies. (c) The total magnetic field derived from the vector components measured by the diamond magnetometer during the deep-sea cruise, showcasing the interference from the submersible thruster. The red line indicates periods when the thruster is operating continuously, and the black line represents it operating infrequently. The electromagnetic noise from the submersible is high due to the close strapdown setup of the magnetometer. (d) The noise density spectrum obtained during the deep-sea cruise, derived from the data in (c).

The frequency-modulation method is employed to modulate the PL signal into higher frequency domains, thereby effectively circumventing the influence of low-frequency electronic noise. When the central frequency of the applied microwave field remains invariant, the dynamic range of the magnetometer is limited by the linewidth of the CW-ODMR spectrum. Consequently, even a slight change in the magnetometer’s orientation within the geomagnetic field could exceed its effective dynamic range. Sweeping the microwave frequency and capturing a complete CW-ODMR spectrum for the center resonant frequency can expand the dynamic range, but the additional time consumption will restrict the sensor’s real-time performance. Here we successfully improved the dynamic range of magnetometry by employing a frequency-locking technique that utilizes a homebuilt field-programmable-gate-array- (FPGA) based PID controller [32,44]. High-speed switching of microwave frequencies and a built-in-hardware feedback algorithm are applied to track the zero-crossing points of the CW-ODMR spectrum, obtaining the resonant frequencies for spin-state transitions. Subsequently, the full-vector magnetic field and temperature information sensed by diamond could be simultaneously obtained from the resonant frequencies. In the functionality test shown in Figs. 3a and 3b, a square-wave magnetic field with an amplitude of $(4.5\ \mu {\text{T}},7.7\ \mu {\text{T}}, 5.3\ \mu {\text{T}})$ is applied to the magnetometer, showcasing the full-vector measurement capability of the diamond magnetometer. The specific design and operating details of the sensor system are provided in the supplementary materials (SM).

## RESULTS

### Experiment environment

The deep-sea magnetometry experiments were carried out using a 4500-m-level manned submersible during the TS2-18-7 cruise. The experiments were executed at two sites near the Haima Cold Seep. In each experiment, the diamond quantum magnetometer carried by the submersible descended to a depth of approximately $1300\ \text{m}$, and performed magnetometry measurements above the seafloor. The environmental temperature and pressure data of the sensor are listed in SM Fig. S2.

### Magnetometer performance

During the experiments, we conducted an in-field compensation experiment (see SM) and assessed the diamond sensor's measurement capabilities both on land and at the sea-bottom. After the magnetometer was deployed on the submersible and descended to the target depth, the initial test conducted was the verification of the magnetic noise measurement, with the results showcased in Figs. 3c and 3d. However, due to the magnetometer’s close strapdown configuration near the submersible’s electrical system, the electromagnetic interference from the submersible itself could not be completely eliminated.

The diamond magnetometer conducted the measurement of the submersible’s background static magnetic noise by deactivating the operational electronic devices and maintained quiescent at the bottom of the seafloor. The comparison between the data obtained during the submersible’s static situation and dynamic situation is demonstrated, from which electromagnetic interference from the submersible’s propulsion system and the magnetometer’s realtime capability in different motion attitudes can be observed. The detected magnetic noise levels were $11.4\ \text{nT}/\sqrt{\text{Hz}}$ when the submersible’s electric propulsion engine was activated and $2.0\ \text{nT}/\sqrt{\text{Hz}}$ when it was turned off.

Additionally, the stability and robustness of the diamond magnetometer were qualified during the two 8-h deep-sea diving experiments. The microwave signal could always follow the resonance frequency of the NV centers in continuous attitude change.

### Magnetic compass

**Figure 4.**
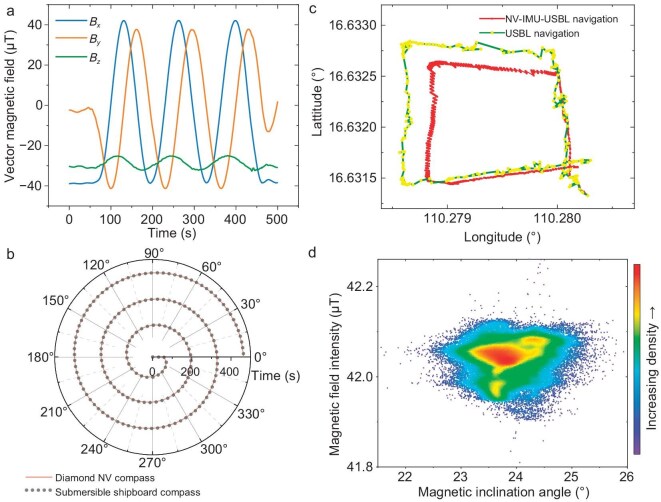
Demonstration of the diamond NV center magnetometer’s application capability in a deep-sea geomagnetic environment. (a) The vector magnetic field measured by the diamond magnetometer when the submersible continuously rotated for three circles at the minimum turning radius. Each line represents a vector component of the measured magnetic field. (b) A comparison between the results of the NV compass and the submersible’s shipboard compass during this rotation maneuvering. (c) The experimental navigation result given by the inertial measurement unit (IMU) system and the diamond magnetometer. The yellow points represent positioning conducted by the USBL system alone, while the red line represents combined navigation positioning carried out jointly by the USBL system, the IMU system and the diamond magnetometer. (d) The geomagnetic inclination and geomagnetic field intensity’s combined distribution recorded by the diamond magnetometer measured at diving site SY523.

The capability of the diamond magnetometer in performing stable and continuous full-vector magnetic field measurements was also validated. This was achieved during the rapidly changing conditions of the geomagnetic field by conducting a 1080$^{\circ }$ turn at the submersible’s minimum turning radius. The successful completion of this maneuver is illustrated in Fig. 4a. The diamond magnetometer accurately recorded the magnetic field during the continuous turning motion of the submersible throughout the cruise, confirming its dynamic range exceeding the geomagnetic field level of $\pm 40\ \mu {\text{T}}$.

Throughout the entire cruise, the diamond magnetometer functioned as a magnetic compass experimentally, and its effectiveness was validated with the built-in magnetic compass on the submersible. As shown in Fig. 4b, during the turning test, the consistency between the yaw angle calculated by the diamond magnetometer and the yaw angle measured by the submersible’s onboard magnetic compass was maintained within 5$^{\circ }$. This test validates the vector measurement capability of the diamond magnetometer in a moving condition. Also, it demonstrates the potential of using the diamond NV center quantum system for navigation tools such as magnetic compasses for the first time.

### Combined navigation

Additionally, by utilizing the vector diamond magnetometer in a deep-sea geomagnetic field, we conduct a short-distance experimental combined navigation demonstration using the diamond magnetometer as an attitude sensor. These results are compared with the submersible’s ultrasonic positioning system, validating the system’s capability to transition from absolute navigation to offline navigation for a short time, as shown in Fig. 4c. The validation experiment of combined navigation based on the ultra-short baseline (USBL) underwater positioning system and the diamond magnetometer was conducted. The yellow points represent positioning conducted by the USBL system alone, while the red line represents combined navigation positioning carried out jointly by the USBL system, the inertial measurement unit (IMU) system and the diamond magnetometer. As the variation of the local magnetic field in the test seafloor area was very small compared to the Earth’s magnetic field, this impact of the geological surroundings on the local magnetic field is neglectable during the navigation. The navigation algorithm was based on an extended Kalman filter (EKF) algorithm with the NV centers’ spin-state information as the main parameters, together with an IMU module to measure the necessary motion data. This method integrates USBL data, IMU data and attitude data from the diamond magnetometer with gravity accelerometer results. The USBL data indicate the submersible’s position, the IMU data provide velocity measurements in the submersible’s body coordinate and the diamond magnetometer provides geomagnetic information for attitude measurement. The vehicle’s state is encapsulated by a six-dimensional vector encompassing position and velocity, and a rotation matrix is computed for each time step, converting raw velocity vectors into a world reference frame. This multimodal data integration enhances the precision of state estimation. The EKF algorithm operates in two primary cycles. The predicted cycle projects the current state estimate forward in time, while the update cycle adjusts this prediction based on new measurements from either the IMU or USBL system, employing a specific measurement matrix for different sensor types.

The navigation was achieved by utilizing the diamond magnetometer as an attitude sensor, thereby showcasing its utility from merely magnetic field measurement to multi-sensor fusion area. This comparison underscores the system’s capability to perform offline navigation for short time intervals, which is useful for underwater vehicle navigation. Because of the presence of constraints such as sensor drift in the IMU system, this experimental navigation system that integrates diamond magnetometers with the IMU system is not sufficient to enable underwater offline navigation for now, resulting in real-time positioning inaccuracies of about $20\ \text{m}$, as demonstrated in Fig. 4c. Looking forward, we aim to further utilize the full-vector measurement capability of diamond magnetometers and incorporate vector geomagnetic navigation into the framework of underwater offline navigation, offering a viable and efficient solution for this realm.

### Geomagnetic measurement

We conducted geomagnetic field measurements at a diving site in the South China Sea with the diamond magnetometer. The geomagnetic inclination angle at the experiment diving site was detected and proven to be consistent with the International Geomagnetic Reference Field (IGRF) model. The observation accords with the IGRF geomagnetic model, and the comparison is shown in Fig. 4d. The geomagnetic information for this area can be calculated using the IGRF model, which predicts a theoretical magnetic inclination of $22.6^\circ$ and a theoretical geomagnetic field strength of $43.3\ \mu {\text{T}}$. Meanwhile, the measured central value of the magnetic inclination $\theta _I$’s distribution was approximately $23.6^\circ$, and the measured central value of the geomagnetic field strength was $42.1\ \mu {\text{T}}$. These results demonstrate the consistency between the geomagnetic data measured by the diamond magnetometer in the deep-sea area and the IGRF model, validating its capability for vector magnetic measurements in geographical environments. The orientation reference for vertical geographic coordinates was provided by a MEMS accelerometer. The geomagnetic data obtained by the diamond magnetometer were processed to obtain the submersible’s heading information.

## CONCLUSIONS

In conclusion, we successfully demonstrated a vector diamond quantum sensor as a deep-sea magnetometer. A compact quantum control and readout system based on the NV centers was developed and optimized to achieve the sensor functionality. The functionality validation was carried out through a series of tests at the 1300-m-depth bottom of the South China Sea, effectively proving the capability of the diamond sensor in comparison to previous shipboard efforts [45–47]. The diamond sensor’s primary functionality to perform simultaneous full-vector magnetic field measurements underwater was successfully demonstrated; it was mounted on a manned deep submersible vehicle and managed to perform electromagnetic disturbance monitoring, experimental navigation and geomagnetic measurement.

We plan to further exploit the diamond sensor’s unique features like extreme pressure resistance in future works. Although the diamond probe itself can resist extreme pressure [26], the auxiliary electronics in this setup cannot currently operate when exposed to the deep-sea environment. A device encapsulation method using pressure-resistant soft materials could protect the auxiliary electronics and further exploit the unique extreme environment compatibility of diamond [48]. This method could extend the diamond magnetometer’s applicability in extreme environments, and give rise to a new *in situ* deep-sea magnetometry.

Beyond the results we demonstrated in this work, the diamond magnetometer still possesses significant potential for performance improvement in sensitivity, calibration-free vector magnetometry [22,42,49,50], chip-level miniaturization based on a micro-nano machining technique, direct deployment in a high-pressure seawater environment [26,51,52] and multiple physical quantity measurements [53–55]. There are also available opportunities for improvement in the sensor deployment method employed in this ocean experiment, considering the electromagnetic interference observed during the expedition. An external rod or distant cable connection could be applied to the diamond sensor for a measurement with less magnetic disturbance [11]. We could expect continuous improvement in the performance of the diamond quantum magnetometer and its deployment in broader fields, creating practical magnetic sensors capable of covering a wide range of application scenarios than traditional magnetometers.

## Supplementary Material

nwae478_Supplemental_File
