# Supplementary material for: Experimental demonstration of a diamond quantum vector magnetometer for deep-sea applications
Source: Natl Sci Rev. 2025 Jan 15;12(4):nwae478. doi: 10.1093/nsr/nwae478 (PMC11992572; doi:10.1093/nsr/nwae478)
Supplement: nwae478_Supplemental_File [file nwae478_supplemental_file.pdf]

RESEARCH ARTICLE

National Science Review
XX:
deep˙sea˙diamond˙magnetometer˙Supplementary˙Material˙Revised,
2024
doi: https://doi.org/xx.xxxx/nsr/XXXX

PHYSICS

Supplementary Material of Experimental Demonstration of Diamond Quantum Vector Magnetometer for Deep-Sea Applications

Ziyun Yu[1,2,†], Yunbin Zhu[1,2,†], Wenzhe Zhang[1,2], Ke Jing[1,2], Shuo Wang[3], Chuanxu Chen[3], Yijin Xie[4,*], Xing Rong[1,2,*] and Jiangfeng Du[1,2,4,*]

ABSTRACT

Keywords: quantum sensing, deep-sea sensor, nitrogen-vacancy center, vector magnetometry

[1]CAS Key Laboratory of Microscale Magnetic Resonance and School of Physical Sciences, University of Science and Technology of China, Hefei 230026, China; [2]Anhui Province Key Laboratory of Scientific Instrument Development and Application, University of Science and Technology of China, Hefei 230026, China; [3]Institute of Deep-sea Science and Engineering, Chinese Academy of Sciences, Sanya 572000, China; [4]Institute of Quantum Sensing and School of Physics, Zhejiang University, Hangzhou 310027, China

*Corresponding authors. Emails: xie1jin@zju.edu.cn xrong@ustc.edu.cn djf@ustc.edu.cn.

Received: XX XX Year; **Revised:** XX XX Year; **Accepted:** XX XX Year

Sensor Design

The sensor is comprised of several functional modules. The detailed internal structure schematic of the sensor is demonstrated in Figure S1, in which the three-dimensional sizes of each functional module have been marked independently. A compact optical setup is developed to initialize and readout the quantum states of the NV centers in diamond. A 520 nm laser diode powered by a homebuilt laser driver is utilized to excite and initialize the spin states. This is followed by a compactly arranged sequence of optical devices for the polarization adjustment, collimation and focus of laser beam. Additionally, a beam splitter is adopted to sample and monitor the excitation laser.

The 1.5 mm × 1.5 mm × 0.3 mm (100)-cut diamond sensor chip is crafted through the chemical vapor deposition (CVD) method, and the NV centers are generated by alternating electron irradiation and annealing process. The geometrics of the diamond is specifically machined for light-trapping total internal reflection (TIR) to improve the excitation efficiency. This design enables more spins to engage in the sensing process, leading to improved sensitivity [1]. A silicon carbide (SiC) plate is displaced on the diamond chip to facilitate heat dissipation and fixation.

To distinguish the NV centers along different orientations, a ringed-type permanent magnet array is used to lift the degeneracy. The PL signal of the NV centers conducts through a compound parabolic concentrator (CPC) and is finally collected by the photodiode.

A homebuilt 4-channel microwave (MW) source is developed to generate the requisite MW field for spin manipulation. Each of the four MW signals is composed of three sub-frequencies at intervals of 2.16 MHz, matching the NV centers' energy-level splitting caused by the hyperfine interaction between the electron spin and the ^{14}N nuclear spin. This approach effectively enhances the utilization efficiency of the spin sensor and the overall signal contrast. The MW source's frequency switching time to ensure stable output signal is about 40 μs, sufficient for the real-time magnetic measurement at 50 Sps sampling rate. The combined MW signal is transmitted through coaxial connection to a broadband radiation structure, and applied to the diamond chip installed on it. A control and readout module based on field programmable gate array (FPGA) takes the role of data acquisition and device control [2]. It consisted of 2 current input channels, one for the fluorescence photocurrent and the other for the reference laser photocurrent, along with a multi-frequency lock-in amplifier and four proportional–integral–derivative (PID) controllers. To track the sensor's motion and attitude, a MEMS inertial measurement unit (IMU) is adopted as affiliated sensor. These system components are arranged on an aluminium alloy framework and installed inside a cylindrical watertight compartment made from titanium alloy. To ensure the watertightness and mechanical safety of the sensor mounted outside the manned cabin of the submersible, a pressure test with

© The Author(s) 2024. Published by Oxford University Press on behalf of China Science Publishing & Media Ltd. This is an Open Access article distributed under the terms of the Creative Commons Attribution License (http://creativecommons.org/licenses/by/4.0/), which permits unrestricted reuse, distribution, and reproduction in any medium, provided the original work is properly cited.

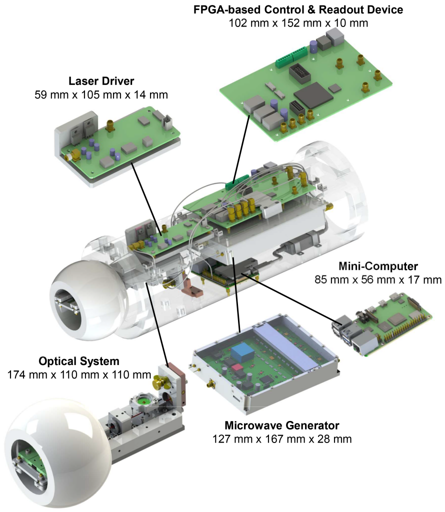

Fig. S1. Detailed schematic figure of the diamond deep-sea magnetometer's internal structure.

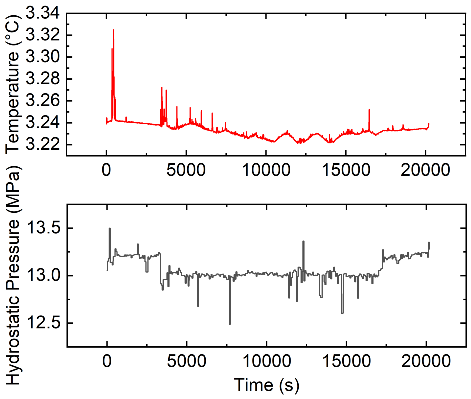

Fig. S2. Environmental information acquired near the seafloor. The upper plot demonstrates the temperature data measured by the submersible's thermometer near the seafloor during the test, and the lower plot demonstrates the hydrostatic pressure data near the seafloor during the test. The pressure data was inferred from the depth gauge of the submersible with the TEOS-10 toolkit.

45 MPa (equivalent to the hydrostatic pressure of 4500 m ocean depth, the operational maximum dive depth of the submersible) was carried out in a high-pressure tank at the Institute of Deep-Sea Science and Engineering, Chinese Academy of Science. Insulation treatment of the sensor prevents the corrosion from seawater and electric leakage, and the residual magnetization of the sensor's titanium alloy compartment is also measured, which is less than 0.1nT.

The diamond sensor is configured to collect full-vector magnetic data and temperature data at 50 Sps for the experiment, and the affiliated IMU sensor operated at 425 Sps. The diamond magnetometer responses to the changes in resonant frequencies caused by magnetic field variation through the implementation of a PID-based MW frequency-locking algorithm. The environmental magnetic field is simultaneously calculated based on the Hamiltonian of NV center. The synchronization of data streams is ensured by a timestamp alignment algorithm based on multi-sensor data correlation and interpolation method. Pre-calibrated results and algorithms are configured in the embedded computer in the form of linear response coefficients, enabling real-time temperature compensation. The entire system consumes about 70 watts of power. As depicted in Figure S2, the environmental pressure near seafloor was about 13 MPa, the environmental temperature data was about 3°C, the internal pressure of the integrated sensor was approximately equal to the standard atmospheric pressure. Meanwhile, the temperature inside integrated sensor was about 26°C.

A watertight cable established the connection between the sensor and the submersible, allowing the crew in the submersible to utilize the magnetometer's data in real-time . To mitigate the magnetic noise from the submersible, a synthetic magnetic compensation algorithm was developed and implemented during the cruise by executing the corresponding calibration maneuvering.

Comparative Experiment

The diamond magnetometer performs full-vector magnetic field measurement based on a single sensor. The atomic vapor cell magnetometer is another type of spin-based quantum sensor, which performs scalar measurement with high sensitivity and has been widely used in marine sensing. To better showcase the differences between these two magnetometers and demonstrate the feature of diamond magnetometer, We performed a comparative experiment between our diamond magnetometer and a commercial atomic vapor cell magnetometer (G-882, Geometrics Inc.). The diamond magnetometer and atomic vapor cell magnetometer were set together on an underwater pod towed by a ship. When the pod rotates and the sensors' attitudes change, the atomic vapor cell magnetometer occasionally encounters the dead-zone and loses its scalar output, while the diamond magnetometer gives stable full-vector output. The test data is shown in Figure S3. The result clearly exemplifies the features of diamond quantum magnetometer to perform full-vector measurements without dead-zone. Researches on atomic vapor cell magnetometers have developed some setups

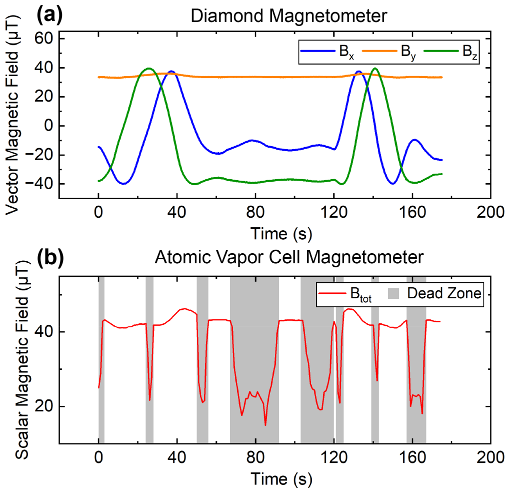

Fig. S3. Experimental comparison of the two magnetometers. (a) The full-vector magnetic field measured by the diamond magnetometer. (b) The scalar magnetic field measured by the atomic vapor cell magnetometer.

in laboratory to achieve similar capabilities with increased system complexity [3,4], but they have not been used in applications like marine sensing.

Real-Time Compensation

The full-vector magnetic field measured by the diamond magnetometer is effectively corrected through real-time calibration with the submersible in motion. During the experiments, continuous and irregular change in submersible attitude is observed due to the influence of ocean currents and submersible movement. The geomagnetic field's projection along the diamond sensor's crystal axis varies rapidly. In this situation, the local magnetic field generated by the submersible could lead to a significant heading error and affect the accuracy of scalar magnetic field measurement.

To obtain a comprehensive and accurate representation of the environmental magnetic field, and to mitigate the influence of the permanent magnetic background stemming from the submersible's magnetization, a method of magnetic compensation is developed and applied. The magnetic interference model is established and solved by stochastic gradient descent (SGD) algorithm, so a real-time calibration could be carried out practically in working environment. The compensated vector magnetic field is presented in Figure S4(a), while the compensated total magnetic field is displayed in Figure S4(b). The submersible's orientation sequentially shifted to the geological directions of north, east, south, and west. A calibration maneuver was executed along a closed square path with 140 m length of side, consisting of pitch, roll and yaw movements, as showcased in Figure S4(c). Figure S4(d) defines the coordinate systems for the submersible and the diamond magnetometer in terms of their respective body frames. By employing the dynamic magnetic field compensation algorithm, the compensated total magnetic field is successfully reduced from tens of μT level to 38 nT, essentially ensuring the magnetometer's applicability. The vector magnetic field data measured by the diamond magnetometer under Earth's magnetic field can be utilized to compensate the magnetic interference corresponding with the error model. The submersible surfaced to 100 m height from the seafloor during the maneuver, to avoid the potentially non-uniform background magnetic field caused by geological surroundings. The acquired data is processed using a modified Tolles-Lawson algorithm to compensate the total field derived from vector magnetic field components [5,6].

Data Collection

The submersible is equipped with built-in motion sensor, navigation magnetic compass, and an ultra-short baseline (USBL) system for relative position from the research vessel Tansuo-2. These built-in sensor data are transported from the submersible to the vessel Tansuo-2 through underwater ultrasonic communication technique at a relatively low sampling rate about 2 Sps, and their system clock are synchronized using the same method. The diamond sensor's system clock is manually synchronized with the submersible's clock at the start of each diving. The sensor's output data is sent to an industrial computer inside the submersible. A series of pre-calibrated parameters of the diamond sensor are used to solve the magnetic field and temperature from these data in realtime. The submersible's pilot could run the compensation algorithm and apply the solved parameters to these preliminary results right after the execution of calibration maneuvering. The initialization of the diamond sensor is conducted on board before the submersible is released from Tansuo-2, verifying its capability to measure throughout the entire deployment process of the submersible with intense attitude changes. A fast-scanning algorithm is developed to initially measure the center frequencies of the ODMR spectrums under varying magnetic field in dynamic situation.

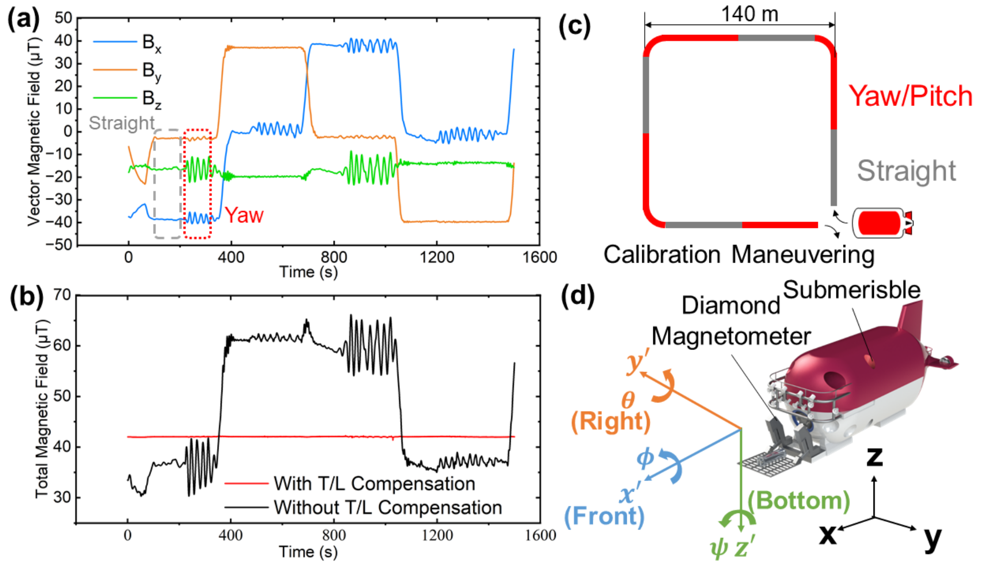

Fig. S4. The in-field compensation process and result of the diamond magnetometer during the deep-sea cruise. (a) The full-vector magnetic field measured during the compensation process. Each component is denoted in a different color. The red dashed box and the black dashed box respectively indicate the yaw/pitch process of the calibration maneuvering. (b)The scalar total magnetic field derived from full-vector magnetic field components with and without the Tolles-Lawson magnetic compensation algorithm. Notably, the algorithm succeeded in reducing the standard deviation of the total magnetic field to approximately 38 nT. (c) The diagrammatic representation of the deep-sea compensation maneuvering. It depicts the submersible navigating through four geological directions and performing a series of straight-yaw maneuvers in sequence. (d) The setup and coordinates of the diamond magnetometer in relation to the submersible. The $\mathrm{x, y, z}$ coordinates represent the body coordinates of the diamond magnetometer, whereas the $\mathrm{x', y', z'}$ coordinates correspond to the submersible's body coordinate system.

REFERENCES

1. Clevenson H, Trusheim ME, Teale C *et al.* Broadband magnetometry and temperature sensing with a light-trapping diamond waveguide. *Nature Physics* 2015; **11**: 393–397.
2. Tong Y, Zhang W, Qin X *et al.* A customized control and readout device for vector magnetometers based on nitrogen-vacancy centers. *Review of Scientific Instruments* 2023; **94**: 014709.
3. Huang H, Dong H, Hu X *et al.* Three-axis atomic magnetometer based on spin precession modulation. *Applied Physics Letters* 2015; **107**.
4. Ben-Kish A and Romalis M. Dead-zone-free atomic magnetometry with simultaneous excitation of orientation and alignment resonances. *Physical review letters* 2010; **105**: 193601.
5. Tolles WE and Lawson J. Magnetic compensation of mad equipped aircraft. *Airborne Instruments Lab. Inc., Mineola, NY, Rept* 1950; 201–1.
6. Bloomer S, Kowalczyk P, Williams J *et al.* Compensation of magnetic data for autonomous underwater vehicle mapping surveys. *2014 IEEE/OES Autonomous Underwater Vehicles (AUV)* (2014) 1–4.
